# Impact of immune checkpoint molecules on FoxP3^+^ Treg cells and related cytokines in patients with acute and chronic brucellosis

**DOI:** 10.1186/s12879-021-06730-3

**Published:** 2021-09-30

**Authors:** Hua-Li Sun, Xiu-Fang Du, Yun-Xia Tang, Guo-Qiang Li, Si-Yuan Yang, Ling-Hang Wang, Xing-Wang Li, Cheng-Jie Ma, Rong-Meng Jiang

**Affiliations:** 1 Department of Infectious Diseases, The Affiliated Hospital of Qingdao University, Qingdao, Shandong China; 2 Department of Infectious Diseases, The Third People’s Hospital of Linfen City, Linfen, Shanxi China; 3 The Laboratory of Infectious Diseases Centre, Beijing Ditan Hospital, Capital Medical University, Beijing, China; 4 Department of Laboratory Medicine, The Third People’s Hospital of Linfen City, Linfen, Shanxi China; 5 Center for Infectious Diseases, Beijing Ditan Hospital, Capital Medical University, Beijing, China

**Keywords:** Brucellosis, Acute infection, Chronic infection, Cytokine, Tregs, PD-1, CTLA-4, GITR

## Abstract

**Background:**

The immunoregulatory functions of regulatory T cells (Tregs) in the development and progression of some chronic infectious diseases are mediated by immune checkpoint molecules and immunosuppressive cytokines. However, little is known about the immunosuppressive functions of Tregs in human brucellosis, which is a major burden in low-income countries. In this study, expressions of immune checkpoint molecules and Treg-related cytokines in patients with acute and chronic *Brucella* infection were evaluated to explore their impact at different stages of infection.

**Methods:**

Forty patients with acute brucellosis and 19 patients with chronic brucellosis admitted to the Third People’s Hospital of Linfen in Shanxi Province between August 2016 and November 2017 were enrolled. Serum and peripheral blood mononuclear cells were isolated from patients before antibiotic treatment and from 30 healthy subjects. The frequency of Tregs (CD4^+^ CD25^+^ FoxP3^+^ T cells) and expression of CTLA-4, GITR, and PD-1 on Treg cells were detected by flow cytometry. Levels of Treg-related cytokines, including IL-35, TGF-β1, and IL-10, were measured by customised multiplex cytokine assays using the Luminex platform.

**Results:**

The frequency of Tregs was higher in chronic patients than in healthy controls (*P* = 0.026) and acute patients (*P* = 0.042); The frequency of CTLA-4^+^ Tregs in chronic patients was significantly higher than that in healthy controls (*P* = 0.011). The frequencies of GITR^+^ and PD-1^+^ Tregs were significantly higher in acute and chronic patients than in healthy controls (*P* < 0.05), with no significant difference between the acute and chronic groups (all *P* > 0.05). Serum TGF-β1 levels were higher in chronic patients (*P* = 0.029) and serum IL-10 levels were higher in acute patients (*P* = 0.033) than in healthy controls. We detected weak correlations between serum TGF-β1 levels and the frequencies of Tregs (*R* = 0.309, *P* = 0.031) and CTLA-4^+^ Tregs (*R* = 0.302, *P* = 0.035).

**Conclusions:**

Treg cell immunity is involved in the chronicity of *Brucella* infection and indicates the implication of Tregs in the prognosis of brucellosis. CTLA-4 and TGF-β1 may contribute to Tregs-mediated immunosuppression in the chronic infection stage of a *Brucella* infection.

## Background

Brucellosis is an important public health problem worldwide, disproportionately affecting poor people living in the least developed and developing countries and infecting more than half a million individuals each year [1]. In China, new infections continue to increase with the dynamic growth of animal husbandry, and the prevalence rate has reached 3.2513/100,000 [2]. Human brucellosis, caused by intracellular bacteria belonging to the genus *Brucella*, is considered a febrile illness that can progress to a long-lasting disease with severe complications, including spondylarthritis, meningitis, and endocarditis [3, 4].

Although human brucellosis has a high incidence and easily develops into chronic infection, patient-friendly treatments and effective vaccines are lacking [4, 5]. Current therapeutic strategies (antibiotics) require months of multi-drug treatment, and treatment failure can lead to the reactivation of the disease. Furthermore, there are no reliable markers for prognosis, diagnosis, and follow-up. The mechanisms underlying chronicity are not fully established. In particular, research on the mechanism through which the bacteria evade the host immune response is expected to provide valuable information on the pathogenesis of brucellosis and facilitate the development of new strategies for its treatment and for preventing its progression to the chronic form.

The Th1 immune response is essential for the clearance of *Brucella* infection, and the antimicrobial effects of pro-inflammatory cytokines, including IFN-γ and TNF-α, have been established [6]. However, *Brucella* can subvert the protective immune response and consequently establish a chronic infection [7]. Previous mouse model studies have shown that *Brucella* may modify virulence factors, such as lipopolysaccharide (LPS), proline racemase protein A (PrPA), and toll-like receptor/IL-1R (TIR)-containing protein, which in turn affect the levels of IFN-γ, TNF-α, IL-10, and TGF-β1. This alters the Th1 immune response and leads to bacterial persistence [8–11]. Additionally, *Brucella* can downregulate the immune response by the induction of Tregs [12]. Evidence of Tregs acting as suppressors of the T cell response to brucellosis was initially obtained from murine models, in which CD4^+^ CD25^+^ Tregs increased in the spleen of BALB/c mice and the antibody-mediated depletion of murine CD25^+^ T cells induced *Brucella* elimination from target organs [13]. Similarly, human clinical studies have demonstrated an increased frequency of FoxP3^+^ Tregs in the peripheral blood of patients with brucellosis [14, 15].

Although Tregs confer host protection by preventing excessive inflammation during infection, they might also enable pathogen persistence by restraining effector immune responses [16]. The overexpression of immune checkpoints by Tregs and the secretion of suppressive cytokines, such as IL-10, TGF-β, and IL-35, are the prominent mechanisms of Treg cell-mediated suppression [17–21]. Numerous recent studies have highlighted the contribution of several immune checkpoints, including CTLA-4, GITR, and PD-1, to the control of Treg functions in various disease settings [22–26]. However, induction of immune checkpoints on Tregs has not been described in brucellosis.

In our study, we analysed circulating CD4^+^ CD25^+^ Foxp3^+^ Treg cells and the expression of checkpoint molecules (CTLA-4, PD-1, and GITR) in peripheral CD4^+^ CD25^+^ Foxp3^+^ Tregs by flow cytometry. We also analysed the secretion of inhibitory mediators (IL-10, IL-35, and TGF-β1) using the Luminex platform in patients with acute and chronic brucellosis and in healthy controls.

## Methods

### Study subjects

Patients with acute infection with a disease course of < 3 months (n = 40) and chronic infection with a course of > 6 months (n = 19) were recruited at the Third People’s Hospital of Linfen, Shanxi Province, China from August 2016 to November 2017. Healthy controls (n = 30) from the same area, matched by sex and age, were included. Brucellosis was diagnosed according to epidemiological data and seroagglutination test (SAT) titre ≥ 1:100 (2+) [27]. Patients were excluded if they were pregnant; had evidence of autoimmune diseases; had other acute/chronic renal, liver, or cardiovascular diseases; or received antibiotic treatment during the 3 months prior to the study. Patients treated with immunosuppressants, immunomodulators, and/or other drugs capable of modifying the immune response during the 3 months preceding the study were also excluded.

The study was approved by the Ethics Committee of Beijing Di Tan Hospital, Capital Medical University and was performed in accordance with the Declaration of Helsinki for medical research involving human subjects. All patients and controls provided written informed consent for participation in the study. Table 1 presents the demographic, clinical, and laboratory characteristics of patients with acute and chronic brucellosis.

**Table 1:** Demographic, clinical, and laboratory findings for patients with brucellosis at the time of sample collection

Variable	Acute patients, no. (%) n = 40	Chronic patients, no. (%) n = 19
Sex		
Male	32 (80.0)	16 (84.2)
Female	8 (20.0)	3 (15.8)
Age (years)		
18–30	5 (12.5)	0 (0)
31–45	13 (32.5)	4 (21.1)
46–60	17 (42.5)	11 (57.9)
61–70	5 (12.5)	4 (21.1)
Area		
Urban area	4 (10.0)	0 (0)
Rural area	36 (90.0)	19 (100)
Transmission mechanisms		
Direct contact with infected sheep	37 (92.5)	15 (78.9)
Ingesting undercooked mutton	3 (7.5)	4 (21.1)
Signs and symptoms		
Fever (> 37.3 °C)	37 (92.5)	10 (52.6)
Sweating	37 (92.5)	9 (47.4)
Constitutional symptoms^a^	28 (70.0)	17 (89.5)
Hepatomegaly or splenomegaly	5 (12.5)	2 (10.5)
Myalgia or arthralgia	26 (65.0)	17 (89.5)
Lumbar pain	9 (22.5)	14 (73.7)
Epididymoorchitis	2 (5.0)	0 (0)
Respiratory disorders	3 (7.5)	0 (0)
Laboratory findings		
Hematologic		
WBC > ULN (3.97–9.15) × 10^9^/L]	6 (15.0)	5 (26.3)
NEU-R > ULN [(50–70)%]	16 (40.0)	7 (36.8)
Transaminasemia		
ALT > ULN [40 U/L]	16 (40.0)	9 (47.4)
AST > ULN [40 U/L]	11 (27.5)	6 (31.6)
CRP > 5 mg/L	35 (87.5)	15 (78.9)
ESR^b^	34 (85.0)	14 (73.7)
Disease duration		
Less than 14 days	14 (35.0)	–
Between 15 and 30 days	23 (57.5)	–
Between 31 and 60 days	3 (7.5)	–
Between 6 and 9 months		8 (42.1)
Between 9 and 12 months		6 (31.6)
Between 12 and 15 months		5 (26.3)

*ULN* Upper limit of normal

^a^Constitutional symptoms comprise anorexia, malaise, asthenia, weight loss

^b^ESR: Male > 15 mm/h, Female > 20 mm/h

### Sample collection and peripheral blood mononuclear cell isolation

Peripheral venous blood samples were collected from patients with brucellosis before treatment and from healthy subjects. The samples were stored in vacutainers (red top) without anticoagulant and EDTA-containing tubes for serum and peripheral blood mononuclear cell (PBMC) isolation. The serum samples were obtained by centrifugation within 4 h of blood collection, and aliquots were stored at 80 °C until use. PBMCs were isolated from EDTA blood by Ficoll-Paque (Amersham Pharmacia Biotech, Uppsala, Sweden) density gradient centrifugation. Cells were cryopreserved in foetal bovine serum (GIBCO, Grand Island, NY, USA) supplemented with 10% dimethyl sulfoxide (DMSO) and stored in liquid nitrogen.

### Flow cytometry

Cryopreserved PBMCs were used to evaluate cell viability by cell surface and intracellular cytokine staining using samples from 34 acute cases, 15 chronic cases, and 29 healthy controls. Cryopreserved PBMCs were thawed in RPMI 1640 medium (Invitrogen, Carlsbad, CA, USA), washed with phosphate-buffered saline (PBS) containing 1% bovine serum albumin (BSA), and incubated at room temperature for 20 min with the cell viability marker Fixable Viability Stain 510 (BD Biosciences, San Jose, CA, USA).

PBMCs were stained using monoclonal antibodies to determine the expression of Treg markers (CD4, CD25, and FoxP3) and co-expression of CD152/CTLA-4 (cytotoxic T-lymphocyte antigen 4), GITR/CD357 (glucocorticoid-induced tumour necrosis factor receptor), and PD-1/CD279 (programmed cell death protein 1).

Cells were incubated with 5 µL of anti-CD4-PerCP-cy5.5 (BD Biosciences), anti-CD25-PE-Cy7 (BD Biosciences), anti-CD279-BV421 (BD Biosciences), and anti-CD357-Alexa Fluor 488 (eBioscience, San Diego, CA, USA) in the dark for 30 min at room temperature. Intracellular staining for anti-Foxp3-AF647 and anti-CD152-PE was performed using the Pharmingen Transcription Factor Buffer Set, following the manufacturer’s instructions (BD Biosciences). Phenotypic analyses were performed using BD FACS Canto II with Diva (BD Biosciences). Forward scatter and side scatter light gating were used to exclude cell debris. Forward height and forward areas were used to exclude doublet cells. The final analysis was performed using FlowJo 10.0.7 (Tree Star Inc., Ashland, OR, USA). Tregs were defined as CD4^+^, CD25^+^, and FoxP3^+^ cells.

### Cytokine measurement by the multiplex cytokine assay system

Serum cytokine concentrations were measured using a Luminex Flex MAP 3D System (Austin, TX, USA). Three panels manufactured by Millipore Corporation (Billerica, MA, USA) were used to test three cytokines, TGF-β1 (MILLIPLEX MAP TGF-β1 Single Plex Magnetic Bead Kit; Cat. # TGFBMAG-64K-01), IL-10 (MILLIPLEX MAP Human Cytokine/Chemokine Magnetic Bead Panel I; Cat. # HCYTOMAG-60K), and IL-35 (MILLIPLEX MAP Human Cytokine/Chemokine Magnetic Bead Panel IV; Cat. # HCYP4MAG-64K), following the manufacturer’s protocol. Briefly, chemically dyed antibody-bound beads were mixed with either a standard solution or a sample, incubated overnight at 4 °C, washed, and then incubated with a biotinylated detection antibody. After the beads were washed, they were incubated with a streptavidin–phycoerythrin complex. The samples were then washed using a handheld magnet and resuspended in sheath fluid. Finally, the samples were run on the Luminex FLEXMAP 3D^®^ (Austin, TX, USA), and the data were collected and analysed using MILLIPLEX Analyst 5.1 (Luminex). A five-parameter regression formula was used to calculate sample concentrations from the standard curves.

### Statistical analysis

Statistical analyses and the graphical presentation of results were performed using GraphPad Prism 7.0 (San Diego, CA, USA). The results are expressed as medians (Q1–Q3). The one-sample Kolmogorov–Smirnov test was used to determine whether data followed a normal distribution. Nonparametric Kruskal–Wallis tests were used to compare outcomes between groups. Bonferroni correction was applied to correct for multiple comparisons. The significance level was set at 0.017 (0.05/3) to identify differences between groups. Spearman rank correlation tests were performed. Statistical significance was set at *P* < 0.05.

## Results

### Demographic characteristics

The demographic and clinical characteristics of the patients are shown in Table 1. Thirty healthy donors (21 men and 9 women) with a mean age of 40 years (range, 24–60 years) were studied in parallel.

### Upregulation of immune checkpoints on Treg cells in patients with brucellosis

**Fig. 1:**
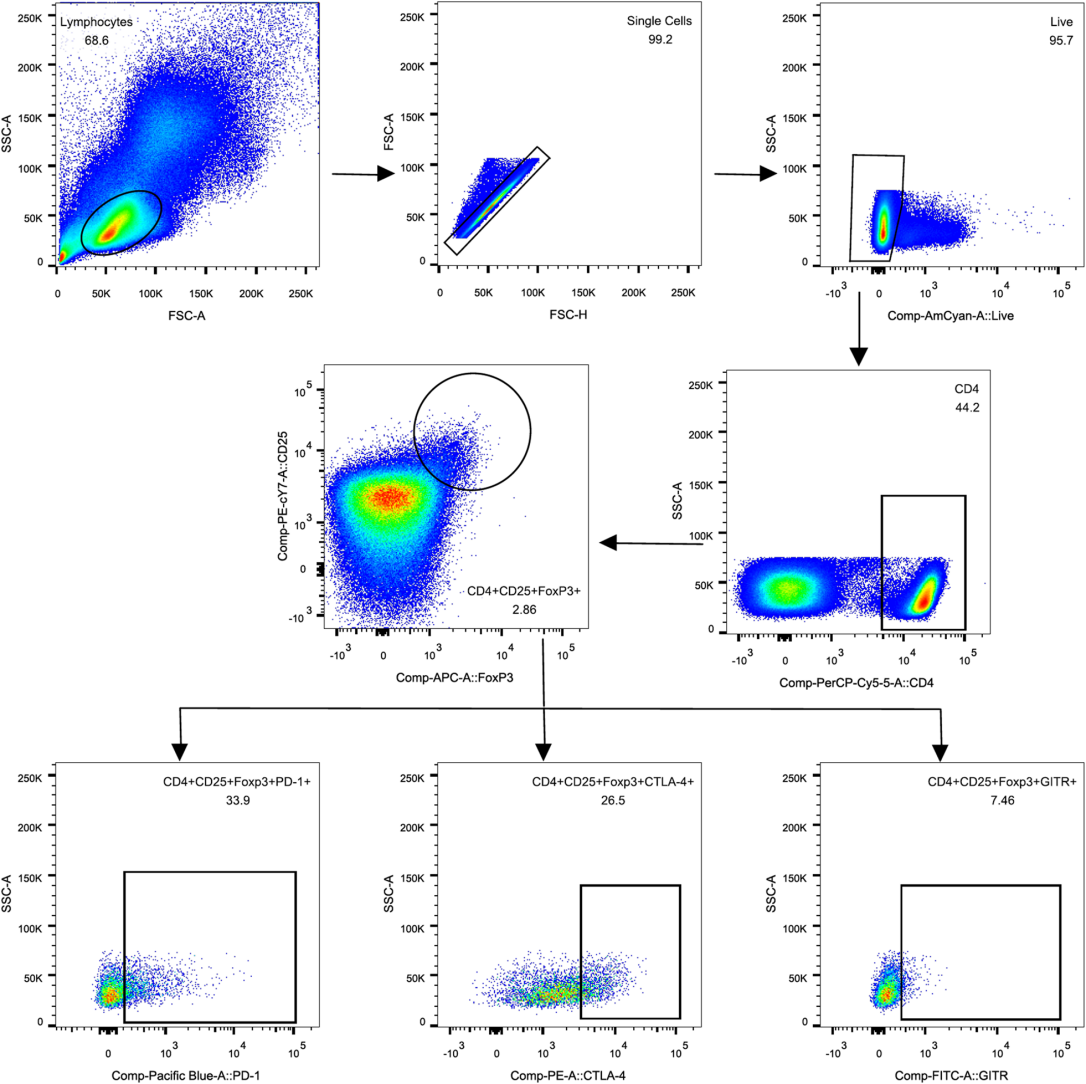
Representative flow cytometry gating strategy for different Treg subsets. In this sample gating, cells were first gated for lymphocytes (SSC-A vs FSC-A). The lymphocytes were gated based on single cells and live cells. Tregs were identified by CD4-positive, CD25-positive, and FoxP3-positive staining and were further analysed for PD-1-positive, CTLA-4-positive, and GITR-positive Treg^+^ T cells

**Fig. 2:**
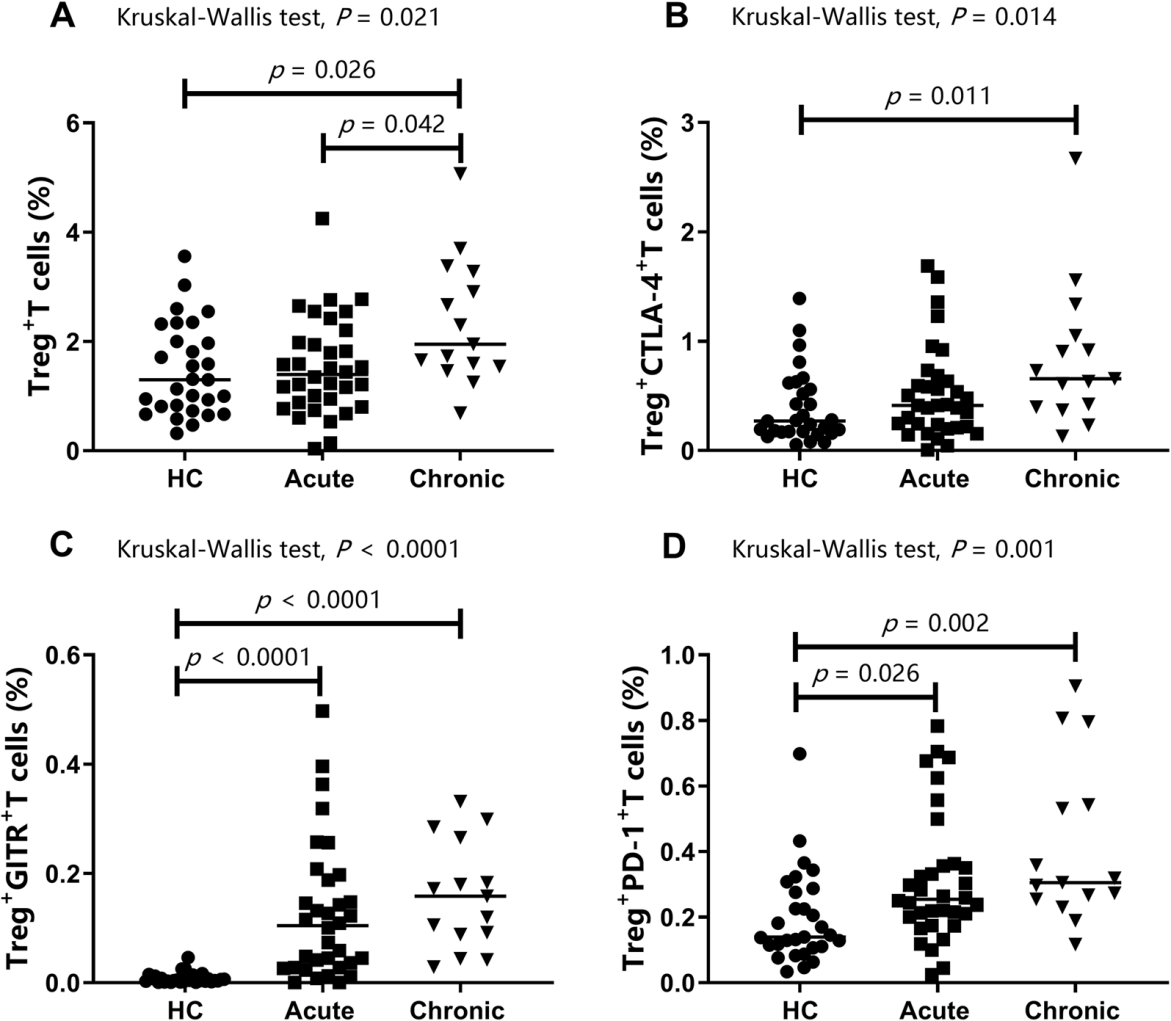
Flow cytometric analysis of Treg cell frequencies and immune checkpoints. CTLA-4, GITR, and PD-1 were evaluated on Treg cells in acute brucellosis (n = 34), chronic brucellosis (n = 15), and HC (n = 29). Results are expressed as percentages of Treg cells (**A**) and percentages of cells expressing (**B**) CTLA-4, (**C**) GITR, and (**D**) PD-1. Percentages (%) are indicated on the *Y*-axis and lines represent median values. Kruskal–Wallis tests were used for comparisons between groups, and Bonferroni correction was applied to compensate for multiple comparisons. *Treg* CD4^+^CD25^+^FoxP3^+^ T cells, *HC* healthy control, *Acute* acute brucellosis, *Chronic* chronic brucellosis

Tregs were identified by flow cytometry as CD4^+^ T cells expressing both CD25 and FoxP3 (Fig. 1). To evaluate the expression of CTLA-4, GITR, and PD-1 on Tregs, we analysed the following subpopulations: CD4^+^ CD25^+^ FoxP3^+^ CTLA-4^+^ T cells (CTLA-4^+^ Tregs), CD4^+^ CD25^+^ FoxP3^+^ PD-1^+^ T cells (PD-1^+^ Tregs), and CD4^+^ CD25^+^ FoxP3^+^ GITR^+^ T cells (GITR^+^ Tregs) in peripheral blood from 34 patients with acute brucellosis, 15 patients with chronic brucellosis, and 29 healthy controls. A representative example of the gating strategy is shown in Fig. 1. As shown in Table 2 and Fig. 2, the frequencies of Tregs and CTLA-4^+^ Tregs were higher in patients with chronic brucellosis than in healthy individuals (Fig. 2A, B; *P* = 0.026 and *P* = 0.011, respectively). Although there was no difference in the frequencies of Tregs between acute patients and healthy controls, the frequencies of Tregs in chronic patients were significantly higher than those in acute patients (Fig. 2A; *P* = 0.042). Interestingly, the frequencies of GITR^+^ Tregs and PD-1^+^ Tregs were both higher in patients with acute and chronic brucellosis than in healthy controls (Fig. 2C, D; all *P* < 0.05) while there was no difference between the two patient groups (all *P* > 0.05).

**Table 2:** Comparison of Treg subsets and serum cytokine level frequencies in brucellosis patients and healthy controls

	Acute patients N = 34	Chronic patients N = 15	Healthy controls N = 29	Kruskal–Wallis test *P*-value^a^
Treg^+^ T cells (%)^b^	1.40 (0.86–2.04)	1.95 (1.54–3.28)*	1.30 (0.77–2.16)	0.021
Treg^+^ CTLA-4^+^ T cells (%)	0.41 (0.22–0.65)	0.66 (0.40–1.05)*	0.27 (0.17–0.59)	0.014
Treg^+^ GITR^+^ T cells (%)	0.105 (0.028–0.190)*	0.159 (0.088–0.265)*	0.004 (0.002–0.010)	< 0.0001
Treg^+^ PD-1^+^ T cells (%)	0.25 (0.19–0.36)*	0.31 (0.25–0.54)*	0.14 (0.11–0.28)	0.001

	N = 40	N = 19	N = 30	
IL-10 (pg/mL)	2.17 (1.30–3.59)*	1.34 (1.02–2.21)	1.29 (0.80–2.34)	0.025
TGF-β1 (pg/mL)	421.2 (112.4–1584.0)	639.3 (200.1–1424.0)*	185.1 (90.9–329.4)	0.016
IL-35 (pg/mL)	0.33 (0.27–0.39)	0.34 (0.29–0.55)	0.35 (0.27–0.65)	0.468

Data are reported as median (Q1–Q3), and Q1 and Q3 represent the first and third quartiles, respectively

*P < 0.05 compared to healthy controls (Bonferroni’s multiple comparisons test)

^a^Kruskal–Wallis tests were used for comparisons between groups (acute patients, chronic patients, and healthy controls)

^b^Tregs, proportion of CD4^+^ CD25^+^ FoxP3^+^ T cells among CD4^+^ cells

### Increased serum IL-10 and TGF-β1 levels in patients with brucellosis

**Fig. 3:**
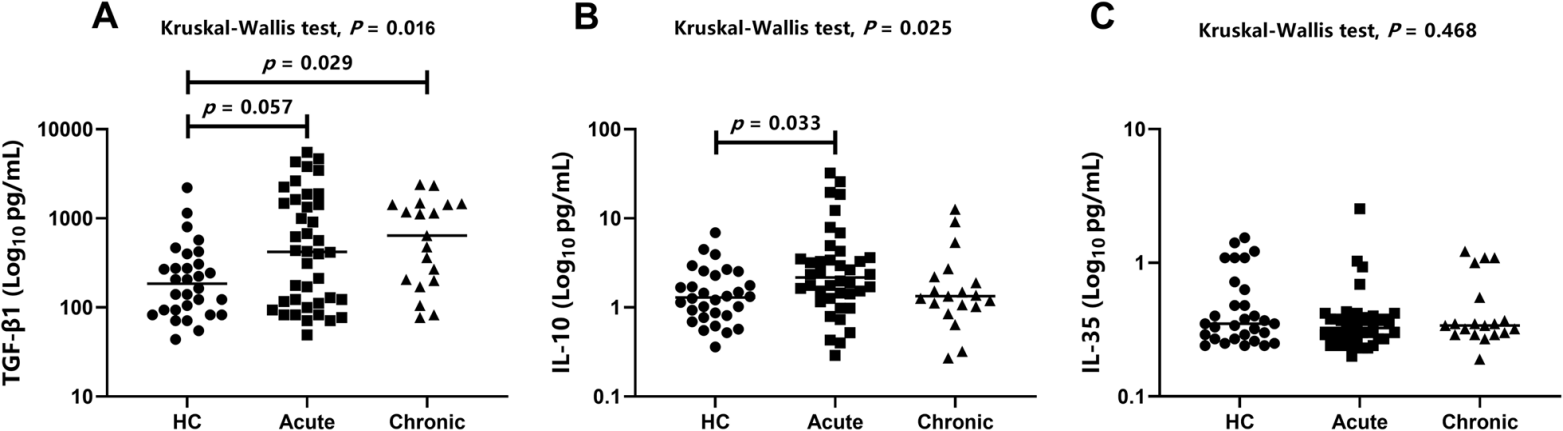
Comparison of cytokine levels among groups. Circulating levels of **A** TGF-β1, **B** IL-10, and **C** IL-35 in serum samples of patients with acute (n = 40) and chronic (n = 19) brucellosis and in healthy controls (n = 30), represented by dots. The horizontal lines in (**A**–**C**) represent the median value. Kruskal–Wallis tests were used for comparisons between groups, and Bonferroni correction was applied to compensate for multiple comparisons. *TGF-β1* transforming growth factor-beta 1, *HC* healthy control, *Acute* acute brucellosis, *Chronic* chronic brucellosis

The key cytokines involved in the immunosuppressive function of Tregs are IL-10, TGF-β, and IL-35. Therefore, we assessed anti-inflammatory cytokines in the serum at different stages of *Brucella* infection, including 40 patients with acute brucellosis, 19 patients with chronic brucellosis, and 30 healthy controls. As shown in Table 2 and Fig. 3, the available data for the three cytokines were compared among the three groups, revealing significant differences in the serum levels of TGF-β1 and IL-10 (Kruskal–Wallis test; *P* = 0.016 and *P* = 0.025, respectively) (Fig. 3A, B) but no significant differences in serum IL-35 levels (Fig. 3C). After applying Bonferroni correction to adjust for multiple comparisons, levels of TGF-β1 (*P* = 0.029) in patients with chronic brucellosis and IL-10 (*P* = 0.033) in patients with acute brucellosis were found to be higher than those in healthy controls (Fig. 3A, B). However, there were no significant differences in serum TGF-β1 and IL-10 levels between patients with acute and chronic brucellosis.

### Correlations between immune parameters

**Fig. 4:**
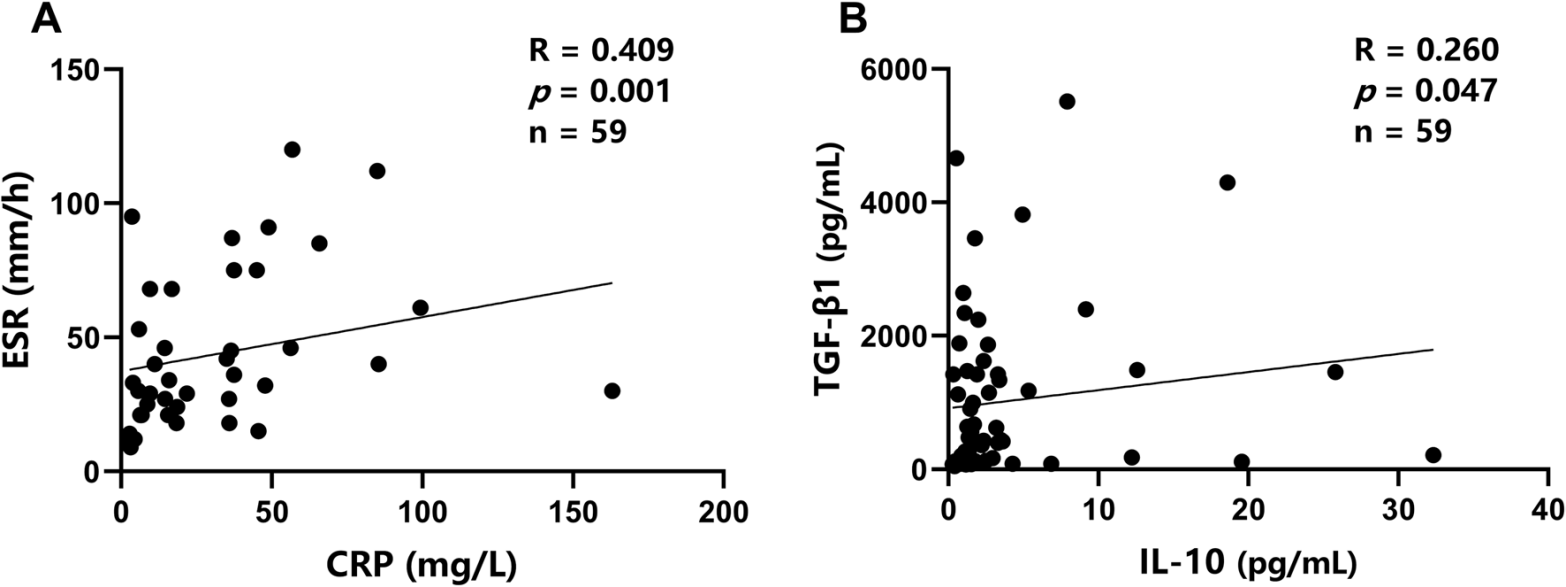
Summary of correlation analyses. Correlations between clinical laboratory parameters CRP and ESR (**A**) and between levels of serum anti-inflammatory cytokines TGF-β1 and IL-10 (**B**) in patients with acute and chronic brucellosis (n = 59). *R*, Spearman correlation coefficient

**Fig. 5:**
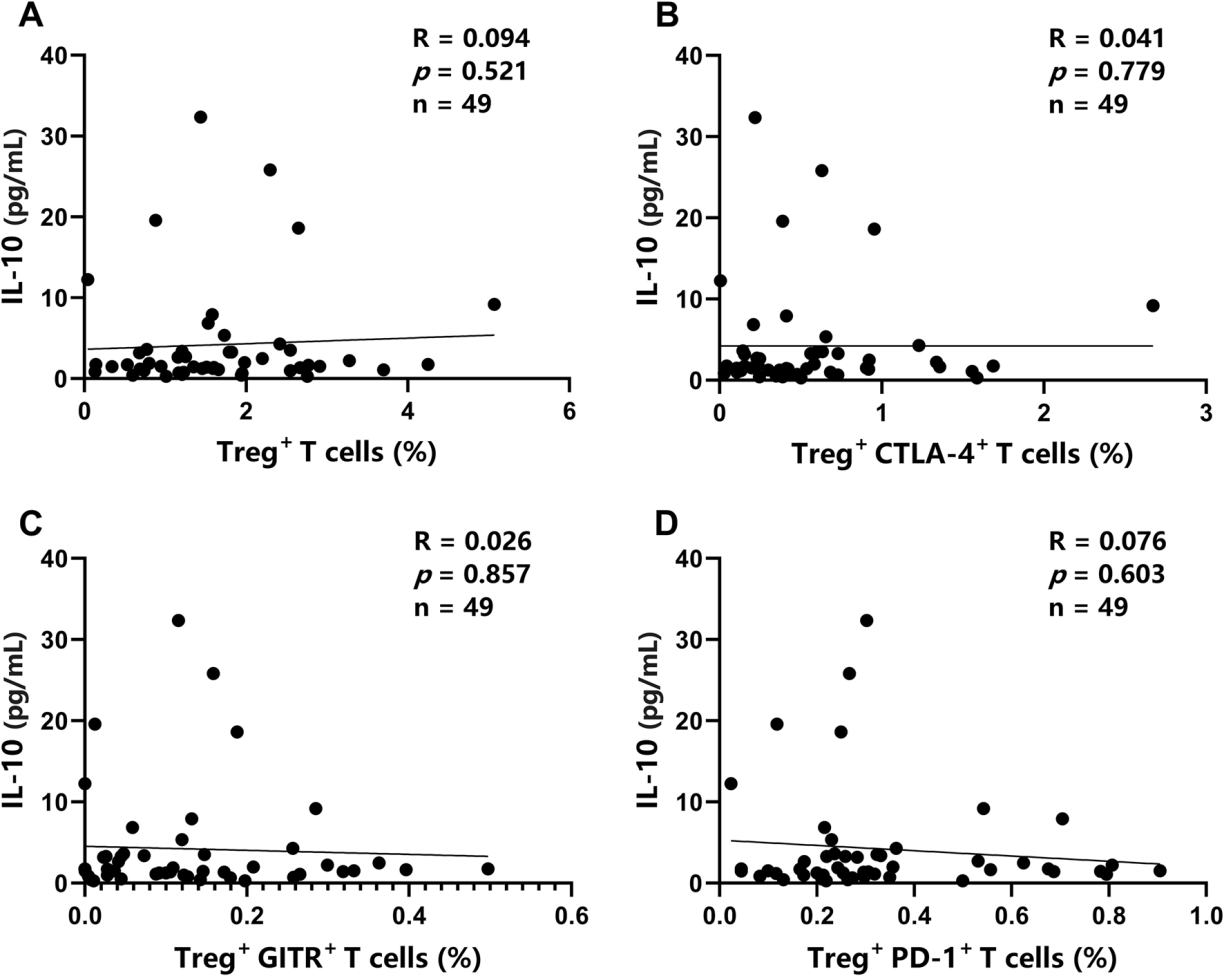
Correlations between serum IL-10 levels and various parameters. Spearman’s correlation coefficient (*R*) was used to evaluate correlations between levels of IL-10 and the frequency of Tregs (**A**) and expression of CTLA-4 (**B**), GITR (**C**), and PD-1 (**D**) on Tregs in acute and chronic brucellosis (n = 49)

**Fig. 6:**
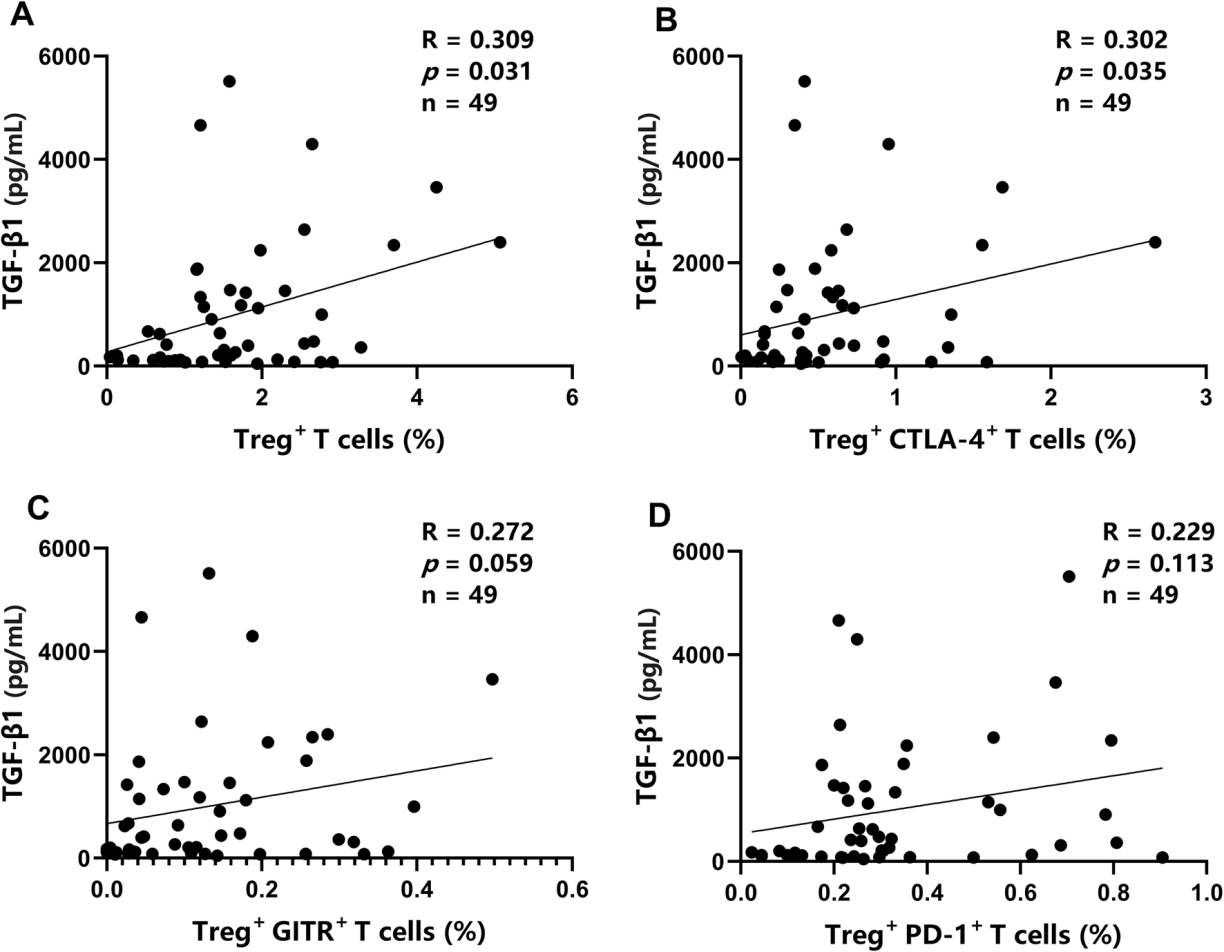
Correlations between serum TGF-β1 and various factors. Spearman’s correlation coefficient (*R*) was used to evaluate correlations between increased serum TGF-β1 levels and the frequency of Tregs (**A**) and expression of CTLA-4 (**B**), GITR (**C**), and PD-1 (**D**) on Tregs in acute and chronic brucellosis (n = 49)

We further evaluated correlations between immune parameters. We detected a weak positive correlation between levels of TGF-β1 and IL-10 in patients with brucellosis (Fig. 4B; *R* = 0.260, *P* = 0.047). C-reactive protein (CRP) was positively correlated with the erythrocyte sedimentation rate (ESR) (Fig. 4A; *R* = 0.409, *P* = 0.001) in patients with brucellosis. No correlations were observed between serum IL-10 levels and immune cell parameters (Fig. 5A–D). Serum TGF-β1 levels were positively correlated with the frequencies of Tregs and CTLA-4^+^ Tregs (Fig. 6A, B; *R* = 0.309, *P* = 0.031 and *R* = 0.302, *P* = 0.035, respectively) but not with the frequencies of GITR^+^ or PD-1^+^ Tregs (Fig. 6C, D).

## Discussion

We investigated the expression of several important immune checkpoint molecules on Tregs and serum levels of Treg-related inhibitory cytokines in acute and chronic patients with brucellosis. Our data agree with previous results showing that *Brucella* may trigger an increase in circulating Tregs in patients with brucellosis [14, 15]. Importantly, CTLA-4 expression was elevated in patients with chronic brucellosis, and PD-1 and GITR were elevated in patients with acute and chronic brucellosis. These findings provide the first evidence that *Brucella* may induce the overexpression of immune checkpoints on Tregs, suggesting that CTLA-4, GITR, and PD-1 may contribute to the impairment of control of *Brucella* infection. Furthermore, in our study, serum IL-10 levels were significantly increased in patients with acute brucellosis, consistent with previous research [28, 29]. Increased serum TGF-β1 levels were positively correlated with Tregs, and CTLA-4 expression on Tregs further supported the critical role of TGF-β1 in mediating the reduced T cell function in patients with brucellosis [30].

Tregs express the transcription factor FoxP3^+^, constitute an essential counterbalance of pro-inflammatory Th1 response, and are required to maintain immune homeostasis. However, the excessive induction of Tregs, as one of the key immune subversion mechanisms, facilitates the development of chronic infections. Few studies have described the association between Foxp3^+^ Tregs and patients with brucellosis. Both acute and chronic patients, particularly chronic patients, have higher proportion of Tregs [14, 15]. Our study also found a higher percentage of Tregs in chronic patients than in acute patients, suggesting that high levels of Tregs may be a risk factor for the development of chronic *Brucella* infections.

Co-signalling immune checkpoint molecules, including co-inhibitory receptors (such as CTLA-4 and PD-1) and co-stimulatory receptors (such as GITR), are defined as ligand–receptor pairs that exert key roles in the suppressive activity of Tregs [31]. CTLA-4 and PD-1 belong to the CD28 superfamily and are highly expressed by activated Tregs, which generate a negative signal upon ligand interactions [23]. Although GITR co-stimulation increases the activation and proliferation of TCR-triggered effector T-lymphocytes, high expression of GITR on FoxP3^+^ Tregs favours the expansion and suppressive capacity of Tregs [32–34]. In this regard, the expression of these immune molecules on Tregs was critically important for their suppressive function. During the last decade, studies have revealed the roles of Treg immune checkpoints in infectious diseases [35, 36]. In malaria-infected individuals, the overexpression of CTLA-4, PD-1, and GITR in T cells is associated with impaired parasite clearance [37–39]. A study on patients with tuberculosis has shown that the frequency of CTLA-4-expressing Treg cells is increased in blood circulation, and CTLA-4 blockade reverses the suppressive effects of Tregs [40]. Our study also found that Tregs in acute and chronic patients showed upregulation of GITR and PD-1 expression, with no difference between the two groups. This novel finding suggests that these inhibitory pathways may contribute to immune dysfunction against brucellosis and that the immunosuppressive state is induced in the early stages of infection [29, 41, 42]. Interestingly, CTLA-4 expression is up-regulated only in chronic patients, suggesting that this inhibitory molecule may be associated with persistent *Brucella* infections. However, owing to the lack of reliable indicators to assess disease severity [43] and relevant data for treating patients, further research on the clinical significance of these signalling molecules is needed.

Tregs may directly inhibit effector T cells by secreting suppressor cytokines, such as IL-10, TGF-β1, and IL-35 [16, 17, 44–46]. In a murine model of brucellosis, IL-10^−^ knockout mice showed reduced bacterial loads in the spleen, and early IL-10 production by CD4^+^ CD25^+^ T cells prevented the immune activation of macrophages and promoted persistent intracellular infection [29, 47]. Consistent with this study, our study also found that serum IL-10 only increased in acute patients, indicating that IL-10 may only be involved in the early stage of infection. The immunosuppressive function of TGF-β1 in brucellosis has been partially confirmed in a previous study [30] in which the overproduction of TGF-β1 was detected in the sera of patients with chronic infection. Therefore, further anti-TGF-β antibody may augment the proliferative responses to *Brucella* extract stimulation in PBMCs. Similar results were found in our study, in which only patients in the chronic phase had significantly elevated serum TGF-β1 levels. Meanwhile, further correlation analysis found that increased serum TGF-β1 levels are positively correlated with the percentage of Treg and expression of CTLA-4, further supporting the hypothesis that TGF-β contributes to the modulation of the immune response to *Brucella* infection and may be related to chronic infection. However, further studies are needed to confirm this specific mechanism. Additionally, large-scale and multiple follow-up point studies are needed to determine whether TGF-β1 can be used to monitor treatment effects and assess disease status. Although IL-35 is a recently identified member of the IL-12 family of cytokines [17], it maybe specifically secreted by Tregs and is required for these cells to exert maximal suppressive activity [18]. In our study, we did not observe increased serum IL-35 levels in patients with acute or chronic brucellosis, suggesting that *Brucella* did not induce the production of IL-35 and that the IL-35 signalling pathway was not involved in Treg-mediated immunosuppression.

Most research data on the chronicity mechanism of brucellosis come from animal models, and few data have been produced on human brucellosis. Although we have conducted research on the possible immunological factors of chronic human brucellosis infections, this paper represents only a single exploratory study on this issue, and therefore this study has several limitations. First, we only analysed limited phenotypic characteristics of Tregs; further functional assays of a wider array of immune molecules are required to provide more evidence for immunotherapy in human brucellosis. Second, in addition to Treg cells, other cells secreted low levels of IL-10, TGF-β1, and IL-35. Therefore, immunosuppression mediated by these inhibitory cytokines in human brucellosis was further comprehensively evaluated by in-vitro experiments. Furthermore, due to poor patient compliance, our research did not consider data from follow-up visits of brucellosis patients undergoing treatment.

## Conclusions

Finally, in our study, the proportion of Tregs in chronic patients was higher than that in acute patients and healthy controls. This suggests that Treg cell immunity may be involved in the chronicity of *Brucella* infection and that Tregs could be used as an early risk indicator for brucellosis. Furthermore, increased TGF-β1 production and expression of CTLA-4 in chronic patients and the correlation between serum TGF-β1 and frequencies of Tregs or CTLA-4^+^ Tregs further support the weakened antibacterial immune response in the chronic stage and suggest that CTLA-4 and TGF-β1 may contribute to Tregs-mediated immunosuppression in the *Brucella* chronic infection stage. Further analysis of data from follow-up visits, especially regarding the effect of standardised treatment on the changes in these immune indicators, will help identify potential biomarkers for brucellosis progression and response to therapy.

## Data Availability

Not applicable.

## Abbreviations

Tregs: Regulatory T cells
PD-1: Programmed cell death protein 1
CTLA-4: Cytotoxic T-lymphocyte antigen 4
GITR: Glucocorticoid-induced tumour necrosis factor receptor
TGF-β1: Transforming growth factor-β
IFN-γ: Interferon γ
TNF-α: Tumor necrosis factor alpha
LPS: Lipopolysaccharide
PrPA: Proline racemase protein A
TIR: Toll-like receptor/IL-1 receptor
SAT: Seroagglutination test
PBMC: Peripheral blood mononuclear cell
DMSO: Dimethyl sulfoxide
CRP: C-reactive protein
ESR: Erythrocyte sedimentation rate
Cat. #: Catalogue number
